# Detection and Segmentation of Pelvic Bones Metastases in MRI Images for Patients With Prostate Cancer Based on Deep Learning

**DOI:** 10.3389/fonc.2021.773299

**Published:** 2021-11-29

**Authors:** Xiang Liu, Chao Han, Yingpu Cui, Tingting Xie, Xiaodong Zhang, Xiaoying Wang

**Affiliations:** ^1^ Department of Radiology, Peking University First Hospital, Beijing, China; ^2^ Department of Radiology, Peking University Shenzhen Hospital, Shenzhen, China

**Keywords:** pelvic bones, metastases, prostate cancer, deep learning, magnetic resonance imaging

## Abstract

**Objective:**

To establish and evaluate the 3D U-Net model for automated segmentation and detection of pelvic bone metastases in patients with prostate cancer (PCa) using diffusion-weighted imaging (DWI) and T1 weighted imaging (T1WI) images.

**Methods:**

The model consisted of two 3D U-Net algorithms. A total of 859 patients with clinically suspected or confirmed PCa between January 2017 and December 2020 were enrolled for the first 3D U-Net development of pelvic bony structure segmentation. Then, 334 PCa patients were selected for the model development of bone metastases segmentation. Additionally, 63 patients from January to May 2021 were recruited for the external evaluation of the network. The network was developed using DWI and T1WI images as input. Dice similarity coefficient (DSC), volumetric similarity (VS), and Hausdorff distance (HD) were used to evaluate the segmentation performance. Sensitivity, specificity, and area under the curve (AUC) were used to evaluate the detection performance at the patient level; recall, precision, and F1-score were assessed at the lesion level.

**Results:**

The pelvic bony structures segmentation on DWI and T1WI images had mean DSC and VS values above 0.85, and the HD values were <15 mm. In the testing set, the AUC of the metastases detection at the patient level were 0.85 and 0.80 on DWI and T1WI images. At the lesion level, the F1-score achieved 87.6% and 87.8% concerning metastases detection on DWI and T1WI images, respectively. In the external dataset, the AUC of the model for M-staging was 0.94 and 0.89 on DWI and T1WI images.

**Conclusion:**

The deep learning-based 3D U-Net network yields accurate detection and segmentation of pelvic bone metastases for PCa patients on DWI and T1WI images, which lays a foundation for the whole-body skeletal metastases assessment.

## Introduction

The nature of bone marrow makes it a favorite fertile soil into which prostate tumors incline to colonize and grow (1, 2); up to 84% of patients with advanced prostate cancer (PCa) experience bone metastases (3), and more than 80% PCa patients developed relapse in the bone following treatment of the primary site (4). The mortality of PCa is 6.6-fold for those with bone metastases compared to those without bone metastases (5). Accurate detection and assessment of metastatic burden in bone are of fundamental importance for radiologists.

Bone scintigraphy (BS) and computed tomography (CT) scans were endorsed as the standard imaging method in the staging and follow-up of metastatic PCa (6), while it is gradually clear that the reduced accuracy of BS and CT in the detection and therapeutic response evaluation of bone metastases reduces their effectiveness in therapy management (7). Multiparametric magnetic resonance imaging (mpMRI) is emerging as a powerful alternative for metastatic PCa. One of the main strengths of mpMRI is to achieve a precise evaluation of bone metastasis *via* the incorporation of anatomic [e.g., T1 weighted imaging (T1WI)] and functional imaging sequences [e.g., diffusion-weighted imaging (DWI)] (7, 8). The value of volumetric measurements for assessing treatment response has been increasingly discussed, and the measurements of lesion volume on mpMRI should be undertaken on high-quality T1WI images according to the METastasis Reporting and Data System (MET-RADS) for PCa (9). Additionally, the volume of bone metastasis assessed with DWI was reported to show a correlation with established prognostic biomarkers and is associated with overall survival in metastatic castration-resistant PCa (10). In short, the detection and delineation of metastases and evaluation of volume change concerning disease progression or therapy on DWI and T1WI images are key tasks as part of optimal patient management.

Heavy workload of mpMRI images evaluation can be tiresome for radiologists, hence bearing the risk of missed diagnosis for lesions and leading to decreased sensitivity. The measurements of all the metastatic lesions are time consuming, in particular, if multiple metastases are present. In this context, automated and accurate segmentation of bone metastases would be highly beneficial.

Driven by the rapid growth in computer science, the performance of deep learning is on par with or even outperforms radiologists in visual identification, which can perform automated data-oriented feature extraction and thus learning directly the most relevant feature representation from the input images (11, 12). The U-Net algorithm is one of the most commonly used deep learning-based convolutional neural networks (CNNs) (13), which shows potential in detection, segmentation, and classification of metastatic lesions on MRI images such as brain metastases (14, 15) and liver metastases (16). Concerning the automated bone metastasis analysis using the deep learning technique, the research trend is mainly on BS (17, 18) and single-photon emission computerized tomography (SPECT) images (19, 20); less attention has been paid to the diagnosis of mpMRI (21, 22). To this end, we intend to apply the 3D U-Net (23) algorithm for the segmentation of bone metastases on mpMRI images. For proof-of-concept, we focused on the detection and segmentation of bone metastases in the pelvic area.

## Materials and Methods

This retrospective single-center study was approved by the institutional review board, and written informed consent was waived.

### Patient Cohort

A cohort of 955 consecutive patients who had undergone pelvic mpMRI for either clinically suspected or confirmed PCa between January 2017 and December 2020 was reviewed using our institutional image archiving system. The exclusion criteria were as follows: (1) poor image quality (significant motion artifact or chemical shift artifact), (2) uncomplete MR image set, (3) obvious destruction of bone structure, and (4) patients with a history of pelvic fracture or surgery. Finally, the images from 859 patients were included for the 3D U-Net model development of pelvic bony structures segmentation, including a dataset of patients with PI-RADS score of 1–2 or biopsy-proven benign prostate hyperplasia (dataset 1, n = 349), a dataset of biopsy-proven PCa patients without bone metastases (dataset 2, n = 280), and a dataset of biopsy-proven PCa patients with bone metastases (dataset 3, n = 230).

All three datasets were used to develop a pelvic bony structure segmentation model. Then, a 3D U-Net model for bone metastases segmentation was developed using datasets 2 and 3. The patients with primary malignant bone tumors (such as osteosarcoma and myeloma) or definite benign findings (hemangiomas, bone island) on pelvic bones (n = 27) and patients who underwent PCa treatment (endocrine therapy, chemotherapy, or radiotherapy, n = 149) were excluded. In total, 334 patients were enrolled for the model development, including 168 PCa patients with bone metastases and 166 PCa patients without bone metastases.

**Figure 1:**
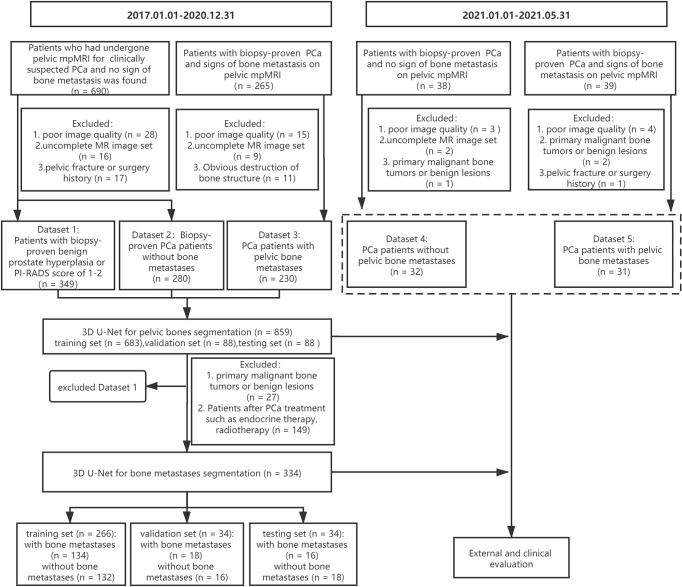
The workflow of data enrollment.

Additionally, 77 patients with biopsy-proven PCa who performed pelvic mpMRI scanning from January 2021 and May 2021 were acquired; according to the above excluding criteria, 63 patients were finally recruited for the external evaluation of the 3D U-Net model including a dataset of 31 PCa patients with bone metastases (dataset 4) and a dataset of 32 PCa patients without bone metastases (dataset 5). The workflow of data enrollment is shown in **Figure 1**.

### Image Acquisition

The pelvic mpMRI acquisitions were performed on three 3.0 T MR units (Achieva, Philips Healthcare; Discovery, GE Healthcare; Interia, Philips Healthcare). The standard pelvic mpMRI protocol at our institution included a T1/T2-weighted sequence, DWI with b-values of 0, 800, or 1,000 s/mm^2^ along with reconstructed ADC images, T1W images obtained using the 2-point Dixon technique with in-phase (T1WI-IP) and out-phase (T1WI-OP), and dynamic contrast-enhanced imaging. DWI images with high b-values (b = 800 or 1,000 s/mm^2^) and T1WI-IP images were selected for PCa bone metastases analyses in this study. Detailed MR imaging parameters of DWI and T1WI-IP sequence are shown in **Table 1**.

**Table 1:** MR imaging parameters of DWI and T1WI-IP sequence.

Sequences		3.0 T Discovery	3.0 T Intera	3.0 T Achieva
DWI	b-value (s/mm^2^)	800	1000	800
	Imaging matrix	256 × 256	240 × 240	156 × 180
	Echo time (ms)	60	78	54
	Repetition time (ms)	4,000	4,959	3,300
	Field of view (mm^2^)	450 × 366	480 × 360	512 × 356
	Section thickness (mm)	8	7	7
	Number of slices	25	28	24
T1WI-IP	Imaging matrix	288 × 192	320 × 200	280 × 180
	Echo time (ms)	2.0	2.4	2.4
	Repetition time (ms)	3.9	7.5	6.7
	Field of view	450 × 360	450 × 350	400 × 400
	Section thickness	4	5	2
	Number of slices	112	112	120
	Bandwidth	166.67	300	450
	Flip angle(°)	13	10	10

T1WI-IP, T1W images obtained using the Dixon technique with in-phase.

### Manual Annotation

The manual annotations were performed with an image segmentation software (ITK-SNAP 3.6; Penn Image Computing and Science Laboratory, Philadelphia, PA). Under the supervision of a board-certified radiology expert (with more than 15 years of reading experience), a radiology resident with 3 years of reading experience evaluated all mpMRI examinations and, section by section, manually annotated eight pelvic bony structures (lumbar vertebra, sacrococcyx, ilium, acetabulum, femoral head, femoral neck, ischium, and pubis) on DWI images and T1WI-IP images. The bone metastases were included in the annotations, which were recognized as bone tissue in this bony structure segmentation model. The manual annotations of the pelvic bony structures were regarded as the reference standard for the 3D U-Net model evaluation.

To establish the reference standard of bone metastases, the radiology resident and expert radiologist conducted a review of the original radiology report and double reviewed the included MR imaging scans and prior/follow-up imaging before annotation. A bone lesion was considered as a metastasis if it showed an MR imaging correlated with adequate image contrast (positive image contrast on DWI images and negative image contrast on those obtained with the T1WI-IP images). The radiology resident performed manual annotations of the metastatic lesions on DWI and T1WI-IP images in a voxel-wise manner (indicated as A1.1). Then, the expert radiologist modified the annotations of A1.1 and the annotations after modification were indicated as A2.1. Both the resident and expert radiologist repeated the annotations and modifications at least 3 weeks later (indicated as A1.2 and A2.2, respectively). The inter- and intraobserver agreement between the manual annotations (A1.1 vs. A2.1; A1.1 vs. A1.2; A2.1 vs. A2.2; and A1.2 vs. A2.2) were estimated using Dice similarity coefficient (DSC).

The bony metastatic lesions in the 31 PCa patients of the external dataset were manually annotated by the resident radiology under the supervision of the expert radiologist, which was taken as the reference standard for external evaluation of the model.

### Model Development

A two-step method for the bone metastases segmentation was proposed using the 3D U-Net model: the first step with a 3D U-Net algorithm for pelvic bone segmentation followed by a second step with a 3D U-Net for bone lesion segmentation within the segmented pelvic bony structures. Both the CNNs were coded by Python3.6, Pytorch 0.4.1, Opencv, Numpy, and SimpleITK, and trained on the GPU NVIDIA Tesla P100 16G.

#### Model Development for Pelvic Bones Segmentation

**Figure 2:**
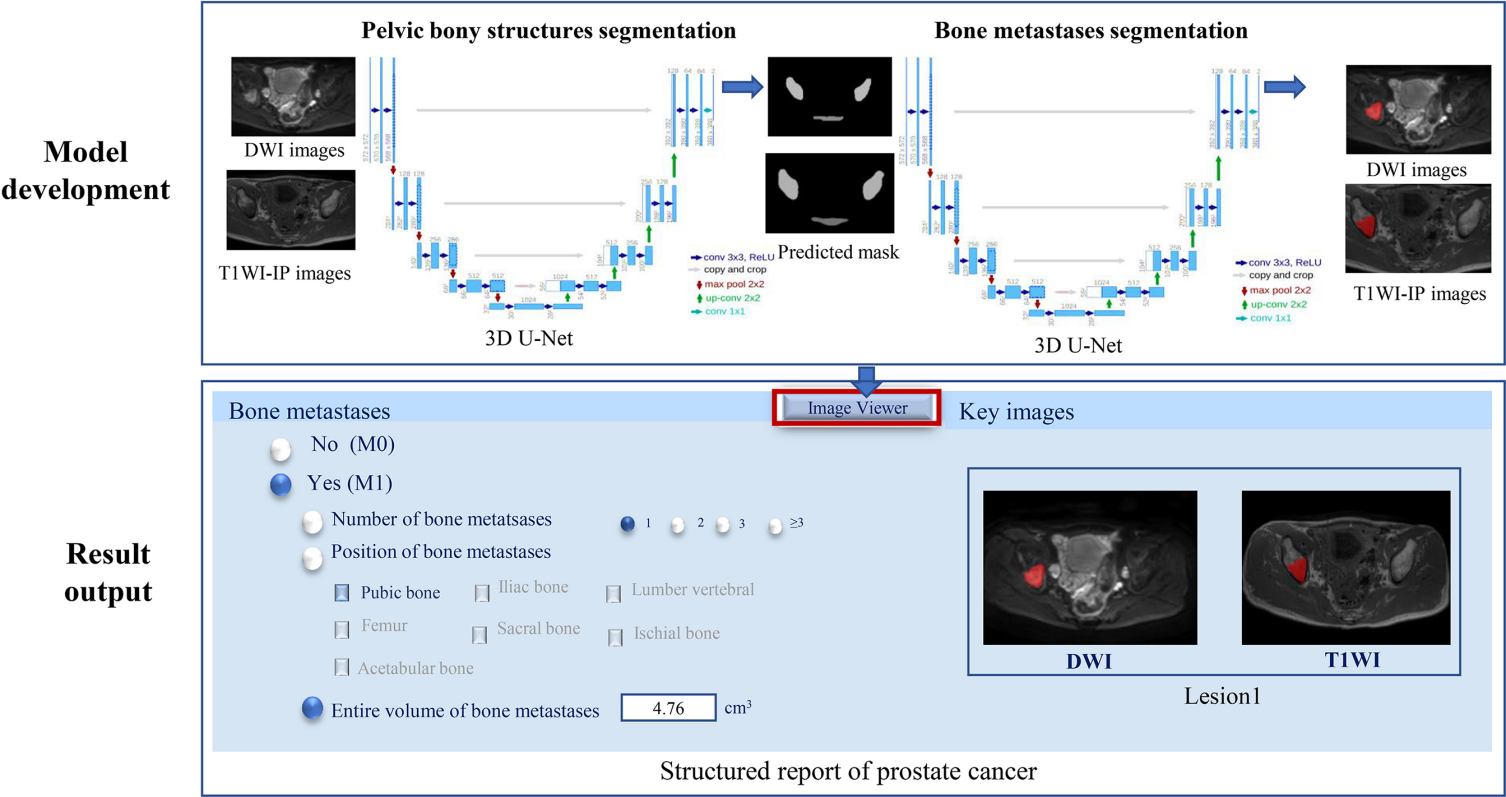
The two-step 3D U-Net for bone metastases segmentation on DWI and T1WI-IP images. T1WI-IP, T1W images obtained using the Dixon technique with in-phase.

The model of the pelvic bony structure segmentation takes the combination of DWI images and T1WI-IP images as input, and each image sequence is used as an independent input data (**Figure 2**). The 859 patients were randomly divided into either training (n = 683), validation (n = 88), or testing (n = 88) sets with a ratio of 8:1:1. During the image preprocessing, the pixel values in images were scaled between 0 and 65,535. Then, the images were resized to 64 × 224 × 224 (z, y, x) by resampling to maintain the optimal image features, and z-score intensity normalization was applied to all images. Skewing (angel: 0–5), shearing (angel: 0–5), and translation (scale: −0.1,0.1) of the images were applied for data augmentation. To remove small spurious segmentation, the two largest connected components of each bone were selected as the final segmentation. A total of 300 epochs of training were performed until validation loss failed to rise. The Adam optimizer was employed to minimize loss with a learning rate of 0.0001, a batch size of 2, and a Dice loss function. Other hyperparameters (such as weight initialization and dropout for regularization) were randomly searched and automatically executed in the validation set during model development.

#### Model Development for Bone Metastases Segmentation

The volume of interest predicted by the model of pelvic bony structure segmentation was used as the mask for the bone metastases segmentation (**Figure 2**). The network configurations were set as follows: training epoch, 250; learning rate, 0.01; batch size, 5; optimizer, Adam optimizer; and loss function, Dice loss.

For post-processing, automatically detected metastases of <0.2 cm^3^ during inference of testing set were regarded as image noise and discarded. The threshold was based on the resolution of T1WI-IP sequences and is determined by referring to the smallest annotated metastases (0.356 cm^3^).

### Model Evaluation

#### Model Evaluation for Pelvic Bony Structure Segmentation

The performance of the network was evaluated by comparing the segmentations generated by the 3D U-Net based on image data from the testing set to the corresponding reference standard represented by the manual segmentations on DWI and T1WI-IP images quantitatively. The evaluation metrics used for the bony structures segmentation include the overlap-based metric (DSC), the volume-based metric [volumetric similarity (VS)], and the spatial distance-based metric [Hausdorff distance (HD)] (24).

#### Model Evaluation for Bone Metastases Segmentation

The performance of the bone metastases segmentation model was evaluated both on detection and segmentation. Detection is defined as the network’s ability to detect a metastasis annotated by the radiologist. One bone metastasis was considered detected when the manual annotation and the predicted segmentation had an overlap >0. Segmentation is defined as its ability to provide a contour identical to that of the radiologist.

The detection performance of the network was quantified at the patient and lesion levels. The sensitivity, specificity, accuracy, positive predictive value (PPV), negative predictive value (NPV), and area under the receiver operating characteristic curve (AUC) were used to assess the performance of the model to discriminate between patients with bone metastases and patients without bone metastases. To determine the detection accuracy of the metastases at the lesion level, we compared the lesions obtained with model predictions and manual annotations to determine the true-positive (TP), false-negative (FN), and false-positive (FP) findings. The recall (correctly detected metastases divided by all metastases contained in reference standard), precision (correctly detected metastases divided by all the detected metastases), and F1 score (harmonic mean of precision and recall) were calculated to assess the detection performance of the model on a lesion-by-lesion basis. In addition, we determined the number of distinct metastatic lesions in each case in the testing set and then divided the data into groups with (a) 1, (b) 2–3, (c) 4–5, and (d) >5 lesions to facilitate subgroup analysis of metastases detection at lesion level.

The metastases segmentation performance of the network was assessed using the metrics of DSC, VS, and HD by comparing the CNN-predicted segmentation and manual segmentation. Besides, the volume of the bone metastases in manual annotations and automated segmentations was calculated to further quantitatively estimate the segmentation efficacy of the U-Net algorithm.

#### Model Evaluation on an External Dataset

The external dataset was used to further assess the efficiency of the model on bone metastases evaluation in the clinical setting. Given the new mpMRI data of PCa patients, the 3D U-Net was supposed to determine the existence of bone metastases (M0 or M1) and output the number, location, and volume of the bone metastases with corresponding segmented masks (**Figure 2**). A bone lesion was considered as being detected if it was segmented on at least one of the two MR imaging sequences (DWI/T1WI-IP). The accuracy of the M-staging of the model was assessed using the receiver operating characteristic curve analysis, and the segmentation performance (DSC, VS, HD) and quantitative measurements (volume) were assessed by comparison with manual annotations.

### Statistical Analysis

MedCalc (version 14.8; MedCalc Software, Ostend, Belgium) and SPSS (version 22.0, IBM Corp., Armonk, NY, USA) were used for the statistical analyses. Numerical data of patients’ age were reported as the mean ± SD (standard deviation), and prostate-specific antigen (PSA) levels were reported as (median, quartile). One-way analysis of variance (ANOVA) was used to compare the characteristics of patients (age, PSA level) among training, validation, and testing sets. The segmentation performance of the algorithm (DSC, VS, and HD) between DWI and T1WI-IP images were compared by paired *t*-test. The McNemar’s test was used to compare the detection performance (sensitivity, specificity, PPV, NPV, recall, and precision) between the two sequences. Bland–Altman analyses were performed to compare manual versus automated bone metastases volume. *p* < 0.05 was considered indicative of a statistically significant difference.

## Results

### Characteristics of Patients

The characteristics of patients are shown in **Tables 2**, **3**. The age and PSA level showed no significant difference among the training, validation, and testing sets on both models (all with *p* > 0.05). The average volume of metastases in the external dataset was 7.39 cm^3^, and no difference was found between the external dataset and model development dataset (*p* = 0.645). Of the 16 PCa patients with bone metastases in the testing set, 2 patients (12.50%) had one metastasis, 5 patients (31.25%) had two to three metastases, 4 patients (25.00%) had four to five metastases, and 5 patients (31.25%) had more than five metastases.

**Table 2:** Characteristics of patients for the pelvic bony structure segmentation model.

Characteristics	Model development (dataset 1 + dataset 2 + dataset 3)				External dataset	*p*-value
	Training set	Validation set	Testing set	*p*-value		
Age (mean ± SD)	68.3 ± 10.5	67.6 ± 10.9	67.8 ± 11.7	0.756	70.7 ± 8.1	0.062
No. of patients	683	88	88	–	68	–
No. of patients with bone metastases	184	23	23	–	34	–
No. of patients without bone metastases	224	16	18	–	34	–
PSA (median, quartile, ng/ml)						
T-PSA	10.49 (7.11, 15.99)	9.73 (8.46, 12.68)	11.19 (7.44, 15.75)	0.556	12.25 (8.89, 26.92)	0.199
F-PSA	1.95 (1.04, 6.71)	2.07 (1.08, 5.78)	2.19 (1.02, 5.46)	0.266	1.65 (1.05, 5.26)	0.112
F/T-PSA	0.12 (0.09, 0.17)	0.10 (0.07, 0.20)	0.10 (0.08, 0.18)	0.587	0.12 (0.09, 0.18)	0.399
Scanners						
3.0 T Discovery	417	56	57	–	31	–
3.0T Achieva	133	17	15	–	13	–
3.0 T Intera	134	15	16	–	24	–

PSA, prostate-specific antigen; T-PSA, total PSA; F-PSA, free PSA; SD, standard deviation.

**Table 3:** Characteristics of patients for the bone metastases model.

Characteristics	Model development (from dataset 2 and dataset 3)				External dataset	*p*-value
	Training set	Validation set	Testing set	*P* value		
Age (mean ± SD)	69.6 ± 10.4	65.9 ± 11.2	68.7 ± 8.9	0.548	70.7 ± 8.1	0.268
No. of patients	266	34	34	–	63	–
No. of patients with bone metastases	134	18	16	–	31	–
No. of patients without bone metastases	132	16	18	–	32	–
PSA (median, quartile, ng/ml)						
T-PSA	13.04 (9.10, 20.1)	12.65 (10.13,18.50)	13.95 (12.95, 23.5)	0.305	12.25 (8.89, 26.92)	0.941
F-PSA	1.29 (1.01,5.41)	1.36 (1.08, 4.38)	1.48 (1.07, 4.73)	0.993	1.65 (1.05, 5.26)	0.091
F/T-PSA	0.07 (0.09, 0.18)	0.09 (0.07, 0.16)	0.09 (0.04, 0.11)	0.356	0.12 (0.09, 0.18)	0.573
Average volume of metastases (median, quartile, cm^3^)	7.50 (5.47, 31.60)	7.98 (2.72, 31.75)	8.05 (2.93, 31.03)	0.945	7.39 (1.23, 28.23)	0.645
No. of metastatic lesions						
1	30 (22.39%)	4 (22.22%)	2 (12.50%)	–	5 (16.13%)	–
2-3	36 (26.86%)	6 (33.33%)	5 (31.25%)	–	6 (19.35%)	–
4-5	24 (17.91%)	4 (22.22%)	4 (25.00%)	–	8 (25.81%)	–
>5	44 (32.84%)	4 (22.22%)	5 (31.25%)	–	12 (38.71%)	–
Total lesions	664	86	89	–	144	–
Scanners						
3.0 T Discovery	172	17	20	–	31	–
3.0T Achieva	71	10	8	–	13	–
3.0 T Intera	23	7	6	–	24	–

PSA, prostate-specific antigen; T-PSA, total PSA; F-PSA, free PSA; SD, standard deviation.

### Assessment of Pelvic Bony Structures Segmentation

**Figure 3:**
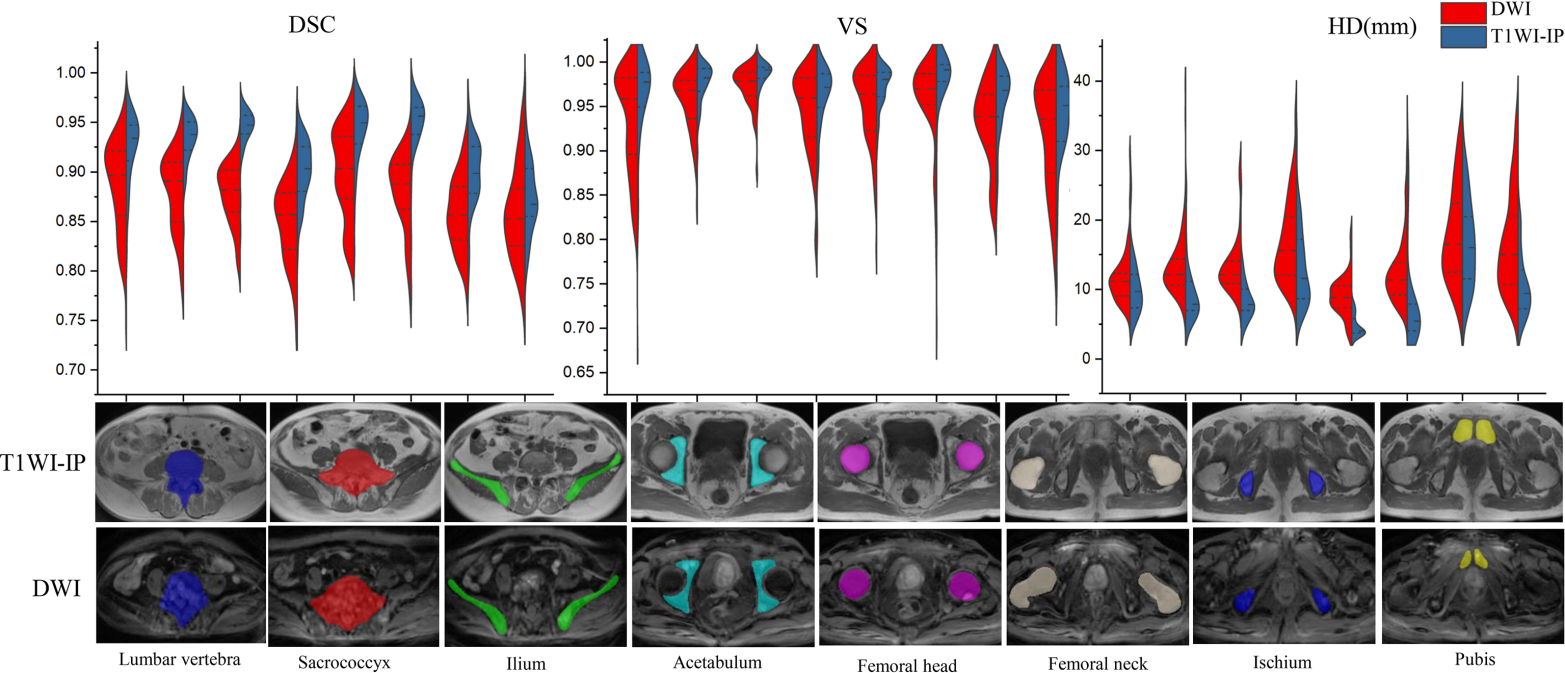
Split violin plots of DSC, VS, and HD (mm) for pelvic bony structures segmentation. DSC, Dice similarity coefficient; HD, Hausdorff distance; T1WI-IP, T1W images obtained using the Dixon technique with in-phase; VS, volumetric similarity.

As shown in **Table 4** and **Figure 3**, in the testing set of pelvic bone segmentation model, the DSC and VS values of eight pelvic bony structures between model prediction and manual annotation are all above 0.85 on both DWI and T1WI-IP images, while the mean DSC and VS values on T1WI-IP images are significantly higher than those on DWI images (all with *p* < 0.05), and the HD is significantly lower. This may be explained by the higher spatial resolution of the T1WI-IP images. Additionally, as detailed in the Supplementary materials (**Supplementary Tables S1–S4**), no significant difference was found among the patients from different datasets (dataset 1 vs. dataset 2 vs. dataset 3) and different scanners (3.0 T Discovery vs. 3.0 T Achieva vs. 3.0 T Intera) on both DWI and T1WI-IP images.

**Table 4:** Segmentation performance of pelvic bony structures.

Bony structures	DSC		*p*-value	VS		*p*-value	HD (mm)		*p-*value
	DWI	T1WI-IP		DWI	T1WI-IP		DWI	T1WI-IP	
Lumbar vertebra	0.89 ± 0.05	0.93 ± 0.03	0.001	0.94 ± 0.06	0.96 ± 0.06	0.034	11.45 ± 3.54	10.63 ± 4.66	0.258
Sacrococcyx	0.88 ± 0.04	0.93 ± 0.02	0.001	0.96 ± 0.03	0.98 ± 0.02	0.001	13.36 ± 4.79	9.56 ± 4.53	0.001
Ilium	0.88 ± 0.03	0.94 ± 0.02	0.001	0.97 ± 0.02	0.99 ± 0.02	0.001	13.34 ± 4.15	8.50 ± 3.30	0.001
Acetabulum	0.85 ± 0.04	0.90 ± 0.03	0.001	0.94 ± 0.05	0.96 ± 0.04	0.017	14.95 ± 6.04	10.17 ± 5.60	0.001
Femoral head	0.90 ± 0.04	0.94 ± 0.03	0.001	0.95 ± 0.04	0.97 ± 0.02	0.001	9.00 ± 2.90	4.77 ± 1.51	0.001
Femoral neck	0.88 ± 0.04	0.95 ± 0.03	0.001	0.96 ± 0.04	0.98 ± 0.05	0.015	12.39 ± 4.40	8.50 ± 5.51	0.001
Ischium	0.86 ± 0.04	0.90 ± 0.03	0.001	0.93 ± 0.05	0.96 ± 0.04	0.001	14.88 ± 6.92	14.62 ± 6.27	0.295
Pubis	0.86 ± 0.05	0.88 ± 0.04	0.022	0.92 ± 0.06	0.94 ± 0.05	0.074	14.72 ± 7.08	10.60 ± 4.58	0.001

DSC, Dice similarity coefficient; HD, Hausdorff distance; T1WI-IP, T1W images obtained using the Dixon technique with in-phase; VS, volumetric similarity.

### The Inter- and Intraobserver Agreement of Bone Metastases Annotations

The interobserver agreement of the manual annotations of bone metastases was assessed by calculating the DSC values between A1.1 and A2.1, and A1.2 and A2.2. The intraobserver agreement was assessed by A1.1 vs. A1.2 and A2.1 vs. A2.2. The DSC values on DWI images were as follows: A1.1 vs. A2.1, 0.90 ± 0.08; A1.1 vs. A2.1, 0.91 ± 0.09; A2.1 vs. A2.2, 0.94 ± 0.05; A1.2 vs. A2.2, 0.91 ± 0.08. In T1WI-IP images, the DCS values were as follows: A1.1 vs. A2.1, 0.89 ± 0.09; A1.1 vs. A2.1, 0.90 ± 0.09; A2.1 vs. A2.2, 0.97 ± 0.04; and A1.2 vs. A2.2, 0.92 ± 0.08. The high DSC values between A2.1 vs. A2.2 confirmed the reliability of the manual annotations. A2.2 was regarded as the reference standard for the lesion segmentation model evaluation.

### The Detection Accuracy of Bone Metastases

The detection performance of the CNN on DWI and T1WI-IP images at the patient and lesion levels are shown in **Table 5**. The detection performance of the model on DWI images was better than on T1WI-IP images concerning the values of the evaluation metrics, while no significant difference was found between the two sequences (all with *p* > 0.05). The results of the subgroup analysis of detection accuracy at lesion level in the testing set showed the highest recall and precision values in patients with single metastases, and both the recall and precision were above 80% for few metastases (≤5 metastases) and multiple metastases (>5 metastases).

**Table 5:** Detection accuracy of bone metastases at patient and lesion levels.

Level	Metrics	DWI	T1WI-IP	*p*-value
Patient-level	Sensitivity (%)	87.5 (61.7–98.4)	81.3 (54.4–96.0)	0.847
	Specificity (%)	83.3 (58.6–96.4)	77.8 (52.4–93.6)	0.852
	Accuracy (%)	85.3 (68.9–95.1)	79.4 (62.1–89.9)	0.789
	PPV (%)	82.4 (56.6–96.2)	76.5 (50.1–93.2)	0.847
	NPV (%)	88.2 (63.6–98.6)	82.4 (56.6–96.2)	0.852
	AUC	0.85 (0.69–0.95)	0.80 (0.62–0.91)	0.442
Lesion-level	Recall (%)	91.01 (81/89)	88.76 (79/89)	0.874
	Precision (%)	84.38 (81/96)	86.81 (79/91)	0.857
	F1-score (%)	87.6	87.8	–
Subgroup analysis				
1	Recall (%)	100 (2/2)	100 (2/2)	
	Precision (%)	100 (2/2)	100 (2/2)	
2–3	Recall (%)	92.9 (13/14)	85.7 (12/14)	
	Precision (%)	86.7 (13/15)	85.7 (12/14)	
4–5	Recall (%)	94.7 (18/19)	84.2 (16/19)	
	Precision (%)	85.7 (18/21)	88.9 (16/18)	
>5	Recall (%)	88.9 (48/54)	90.7 (49/54)	
	Precision (%)	82.8 (48/58)	85.9 (49/57)	

AUC, area under the receiver operating characteristic curve; NPV, negative predictive value; PPV, positive predictive value; T1WI-IP, T1W images obtained using the Dixon technique with in-phase.

### The Segmentation Accuracy of Bone Metastases

**Figure 4:**
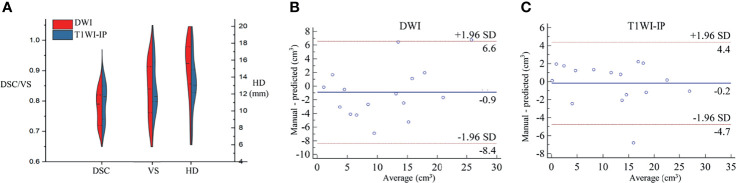
The segmentation accuracy of bone metastases in the testing set. **(A)** Split violin plot of DSC, VS, and HD of the bone metastases on DWI and T1WI-IP images. **(B)** The Bland–Altman plot of the volume difference between manual annotation and model prediction on DWI images. **(C)** The Bland–Altman plot of the volume difference between manual annotation and model prediction on T1WI-IP images. DSC, Dice similarity coefficient; HD, Hausdorff distance; T1WI-IP, T1W images obtained using the Dixon technique with in-phase; VS, volumetric similarity.

The mean DSC, VS, and HD for the automatic metastases segmentation are 0.79 ± 0.05, 0.84 ± 0.09, and 15.05 ± 3.61 mm on DWI images and 0.80 ± 0.06, 0.85 ± 0.08, and 13.39 ± 3.20 mm on T1WI images (**Figure 4A**), which showed no significant difference between the two sequences (*p* = 0.627, 0.741, and 0.175, respectively).

**Figure 5:**
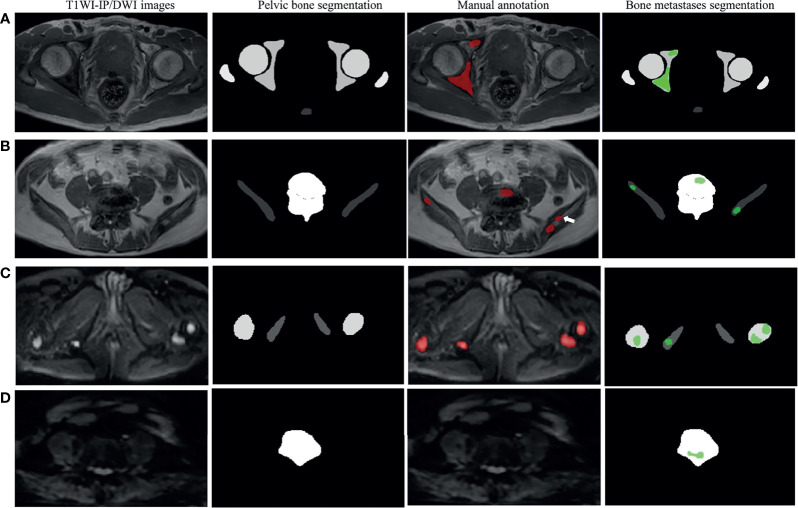
Examples of pelvic bony structure and bone metastases segmentations. **(A)** Two metastases of acetabulum annotated by radiologists were corrected segmented by the model on T1WI-IP images (true positive). **(B)** Four of five metastases annotated by the radiologists were corrected segmented by model on T1WI-IP images; one metastasis on the right ilium was missed (the white arrow pointed, false negative). **(C)** All the four metastases of femoral head and ischium annotated by radiologists were correctly segmented by the model on DWI images (true positive). **(D)** One metastasis of lumbar vertebra was segmented by the model by error, which was not annotated by the radiologists (false positive). T1WI-IP, T1W images obtained using the Dixon technique with in-phase.

The volume differences between manual annotation and model prediction of bone metastases on DWI and T1WI-IP images are shown in **Figures 4B, C**. The limit of agreement (LOA) between the automated and manual segmentation on DWI images was −8.4–6.6 cm^3^ and −4.4–4.4 cm^3^ on T1WI-IP images. Most of the difference values were within the LOA, which showed that the volume of overall metastatic lesions in each patient between manual and automated segmentations agreed closely. Example results of the automatic bone metastases segmentation are shown in **Figure 5**.

### Detection and Segmentation Accuracy on the External Dataset

The sensitivity, specificity, and AUC values of the model in determining the M-staging (M0 or M1) were 93.6% (29/31; 95%CI, 78.6%–99.2%), 93.8% (30/32; 95%CI, 79.6%–99.2%), and 0.94 (95%CI, 0.85–0.98) on DWI images and 87.1% (27/31; 95%CI, 70.2%–96.4%), 90.6% (29/32; 95%CI, 75.0%–98.0%), and 0.89 (95%CI, 0.85–0.98) on T1WI-IP images. The AUC values between the two sequences showed no significant difference (*p* = 0.368).

**Figure 6:**
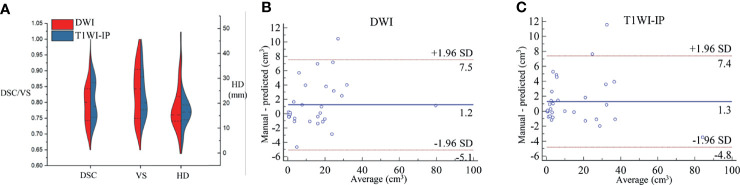
The segmentation accuracy of bone metastases on an external dataset. **(A)** Split violin plot of DSC, VS, and HD of the bone metastases on DWI and T1WI-IP images. **(B)** The Bland–Altman plot of the volume difference between manual annotation and model prediction on DWI images. **(C)** The Bland–Altman plot of the volume difference between manual annotation and model prediction on T1WI-IP images. DSC, Dice similarity coefficient; HD, Hausdorff distance; T1WI-IP, T1W images obtained using the Dixon technique with in-phase; VS, volumetric similarity.

At lesion level, the segmentation accuracy of the model for bone metastases achieved average DSC, VS, and HD values of 0.79 ± 0.06, 0.83 ± 0.08, and 16.03 ± 9.74 mm on DWI images, 0.81 ± 0.06, 0.82 ± 0.07, and 17.20 ± 6.73 mm on T1WI-IP images (**Figure 6A**). The mean volumes of manual annotation and model prediction were 15.35 and 14.10 cm^3^ on DWI images and 15.68 and 14.40 cm^3^ on T1WI-IP images. The volume difference is shown in **Figures 6B, C**.

## Discussion

In this work, we developed a two-step deep learning-based 3D CNN for automated detection and segmentation of bone metastases in PCa patients using whole 3D MR images (DWI and T1WI-IP images), in which the first 3D U-Net focuses on the segmentation of pelvic bony structures and the second one on bone metastases segmentation. On heterogeneous scanner data, the first CNN performed excellent segmentation of pelvic bony structures on both DWI and T1WI-IP images (all with DSC > 0.85), which provides a reliable foundation for the subsequent bone metastases segmentation. Furthermore, our result showed that the proposed CNN provided an AUC of 0.854 and 0.795 on DWI and T1WI-IP images for bone metastases detection at the patient level, and high overlap between automated and manual metastases segmentations was observed (DSC = 0.79 and 0.80 on DWI and T1WI-IP images, respectively). Additionally, by testing on an external dataset, this work demonstrates the CNN’s potential ability of M-staging in clinical practice (with AUC of 0.936 and 0.889 on DWI and T1WI-IP images).

mpMRI has been identified as an essential and crucial imaging modality in PCa diagnosis and metastases evaluation (25, 26). The importance of DWI and T1WI in the detection and quantification of osseous metastasis in patients with PCa has been widely recognized (9, 27). In this study, to avoid the limitation of the application of the CNN if one of these sequences is unavailable, we trained the two-step 3D U-Net CNN using DWI and T1WI-IP images as independent input data. The enrolled participants performed the mpMRI examinations on one of the three different 3.0-T MR scanners with different protocols, and the b-values of the DWI images were different (b = 0, 800 or 0, 1,000 s/mm^2^). In a previous publication (28), we proposed a deep learning-based approach for the segmentation of normal pelvic bony structures. It was the proof-of-concept study for the possibility to detect skeletal metastases located on the pelvic bones. In this study, we used two 3D U-Nets in cascade. The first model was trained to segment the pelvic bony structures. Taking the areas predicted by the first model as the mask, the second model was trained to segment the metastatic lesions on the pelvic bones. The combination of the two 3D U-Nets offers the potential for efficient bone metastases location and quantification. It is important to note that the two-step deep learning model has been widely used to improve the accuracy and stability of the system, such as lymph node detection (29) and PCa segmentation (30).

The high number of FP lesions poses a common drawback in automated detection of metastatic lesions, which has been reported to be approximately seven to eight per scan for brain metastases (31, 32). In the present study, by providing high-quality pelvic bone segmentation masks on DWI and T1WI-IP images, the FP interference from other tissues within the pelvic region (such as metastatic lymph nodes, colon, bladder, etc.) can be effectively eliminated. Moreover, a simple post-processing step was added to avoid FP findings by rejecting all structures with a volume <0.2 cm^3^, which was smaller than the smallest annotated metastases.

Our CNN not only detects almost all metastases but also incorrectly marks other objects as metastases. Most of these FPs were caused by objects that showed a similar radiological appearance to metastatic lesions on DWI and T1WI-IP images. As shown in **Figure 5D**, the high-intensity spinal cord on DWI images within the mask of the lumbar vertebra was detected as metastases by mistake. In addition, the objects that were not or scarcely represented in the training set and thus had an appearance unknown to the network could result in FP as well. These unknown appearances could be other lesions or conditions such as incidental cysts. An inspection of the 15 FP findings on DWI images showed that nine of the FPs were the spinal cord and nerve root structure, and six of the FPs were benign lesions: four cysts and two hemangiomas. The 12 FP objects on T1WI-IP images included eight spinal cord and nerve root structures, three cysts, and one blood vessel structure.

The FN metastases missed by the CNN networks were the small ones, as can be seen in **Figure 5A**, which might be due to the few occupied voxels compared with large metastases. Additionally, on a subgroup analysis, our results suggest that the networks perform well on patients with few metastases (≤5 metastases) and multiple metastases (>5 metastases) in terms of recall and precision, which boosts the clinical utility of the CNN.

Automated segmentation can help radiologists in dealing with an increased number of image interpretations while maintaining high diagnostic accuracy and, simultaneously, may also assist in evaluating treatment response during oncological follow-up. Volumetric assessment proves to be a promising tool for quantification of tumor burden and treatment response evaluation, which is superior to user-dependent conventional linear measurements because metastatic lesions are irregular (33). Compared with manual segmentation, our proposed CNN achieved a high volumetric correlation on both the testing set and the external dataset, which is crucial to help treatment decision-making and potentially improve patient care.

TNM is considered to be one of the most pivotal factors in evaluating the prognosis of PCa, and the existence of bone metastases is a decisive index for the M-staging (34). Concerning M-staging, on the external dataset, our model achieved an AUC of 0.936 (95%CI, 0.845–0.982) on DWI images and 0.889 (95%CI, 0.845–0.982) on T1WI-IP images, which demonstrated that the two-step 3D U-Net algorithm could be used in a clinical context. Besides, the output of the automated segmentation result to the structure report essentially combines visualization, quantification, and segmentation into one step, producing results that can be directly displayed to the radiologists.

U-Net has been proven to possess the potential for bone metastases segmentation. Lin et al. (19) built two deep learning networks based on U-Net and Mask R-CNN to segment hotspots in bone SPECT images for automatic assessment of metastasis. Their results showed that the U-Net-based model achieved better segmentation performance with a precision and recall value of 0.76 and 0.67 than the Mask R-CNN model (precision, 0.72; recall, 0.65). In addition, Chang et al. (35) demonstrated the capability of U-Net in segmenting spinal sclerotic bone metastases on CT images with a Dice score of 0.83. In this study, we explored the feasibility of the 3D U-Net network for pelvic bone metastases segmentation on DWI and T1WI-IP images, and our results further confirmed the segmentation accuracy of the U-Net for bone metastases. However, the comparisons among a couple of other architectures may be helpful to choose an optimal model for metastases segmentation and detection. In the future, we should further explore the performance of other models.

While this study shows high accuracy and performance using CNNs for bone metastases segmentation, several potential study limitations exist. First, the study has a typical drawback of retrospective setting. Testing of the network performance on prospective multicenter data remains a key step towards understanding its clinical value. Second, the relatively small number of patients needs to be noted. Only patients with PCa were included here, which potentially limits the transferability of our CNN to a broad range of bone metastases of other primary tumors (rectal cancer, bladder cancer, etc.). In this context, future studies are needed to evaluate the feasibility of the CNN for bone metastases segmentation of other tumors. Third, in clinical practice, the detection of the lesion by the radiologist is usually done by simultaneous review of anatomical and functional MR images. Besides the Dixon T1WI-IP and DWI images, the Fat or Water images from the Dixon sequence and the short time inversion recovery sequence may also be helpful for the bone metastases evaluation (36, 37). Last, the choice of pelvic examinations as the anatomic target to detect bone metastases and assess the positive–negative status of the patients in terms of metastases is insufficient in clinical practice. The axial and probably whole skeleton, at least from skull to thighs, is necessary, as metastases affect the red marrow-containing areas. Future research is needed to allow for the whole-body bone metastases assessment.

## Conclusion

In summary, our study shows that the deep learning-based 3D U-Net network can automatically detect and segment bone metastases on DWI and T1WI-IP images with high accuracy and thus illustrates the potential use of this technique in a clinically relevant setting.

## Data Availability Statement

The datasets presented in this article are not readily available because the datasets are privately owned by Peking University First Hospital and are not made public. Requests to access the datasets should be directed to wangxiaoying@bjmu.edu.cn.

## Ethics Statement

The studies involving human participants were reviewed and approved by Peking University First Hospital. Written informed consent for participation was not required for this study in accordance with the national legislation and the institutional requirements.

## Author Contributions

XL and XW contributed to the study concept and design. TX and CH contributed to acquisition of data. XL and XW annotated the images data. YC and XZ designed the model and implemented the main algorithm. XL and CH contributed to drafting of the manuscript. All authors contributed to the article and approved the submitted version.

## Funding

This study was supported by Capital’s Funds for Health Improvement and Research (2020-2-40710).

## Conflict of Interest

The authors declare that the research was conducted in the absence of any commercial or financial relationships that could be construed as a potential conflict of interest.

## Publisher’s Note

All claims expressed in this article are solely those of the authors and do not necessarily represent those of their affiliated organizations, or those of the publisher, the editors and the reviewers. Any product that may be evaluated in this article, or claim that may be made by its manufacturer, is not guaranteed or endorsed by the publisher.
