# Supplementary material for: Detection and Segmentation of Pelvic Bones Metastases in MRI Images for Patients With Prostate Cancer Based on Deep Learning
Source: Front Oncol. 2021 Nov 29;11:773299. doi: 10.3389/fonc.2021.773299 (PMC8666439; doi:10.3389/fonc.2021.773299)
Supplement: Supplementary file 1 [file Table_1.docx]

**Supplementary materials**

**Table S1.** Segmentation performance of pelvic bony structures among different datasets on DWI images

| Pelvic bony structures | DSC | | | *P* value | VS | | | *P* value | HD | | | *P* value |
| --- | --- | --- | --- | --- | --- | --- | --- | --- | --- | --- | --- | --- |
|  | S1 | S2 | S3 |  | S1 | S2 | S3 |  | S1 | S2 | S3 |  |
| Lumbar vertebra | 0.87±0.05 | 0.90±0.04 | 0.91±0.04 | 0.061 | 0.93±0.06 | 0.93±0.06 | 0.96±0.05 | 0.102 | 12.00±4.05 | 10.84±2.88 | 10.95±2.90 | 0.361 |
| Sacrococcyx | 0.87±0.04 | 0.88±0.04 | 0.90±0.03 | 0.051 | 0.96±0.03 | 0.95±0.04 | 0.96±0.03 | 0.175 | 13.85±6.18 | 13.02±3.13 | 12.76±2.73 | 0.637 |
| Ilium | 0.87±0.03 | 0.88±0.02 | 0.88±0.04 | 0.323 | 0.97±0.02 | 0.98±0.02 | 0.97±0.03 | 0.178 | 13.25±4.34 | 13.56±4.45 | 13.29±3.60 | 0.959 |
| Acetabulum | 0.86±0.04 | 0.84±0.04 | 0.85±0.05 | 0.426 | 0.95±0.04 | 0.94±0.04 | 0.93±0.06 | 0.213 | 17.08±5.81 | 16.06±6.07 | 17.61±6.62 | 0.683 |
| Femoral head | 0.90±0.04 | 0.92±0.04 | 0.88±0.05 | 0.059 | 0.95±0.04 | 0.96±0.05 | 0.94±0.04 | 0.165 | 8.60±2.30 | 8.56±2.78 | 10.33±3.77 | 0.054 |
| Femoral neck | 0.88±0.03 | 0.89±0.03 | 0.87±0.05 | 0.128 | 0.96±0.04 | 0.96±0.03 | 0.95±0.05 | 0.885 | 11.97±3.94 | 11.51±4.09 | 14.27±5.26 | 0.083 |
| Ischium | 0.86±0.04 | 0.86±0.03 | 0.85±0.04 | 0.647 | 0.94±0.04 | 0.93±0.05 | 0.92±0.06 | 0.522 | 17.55±6.54 | 18.16±6.54 | 18.25±8.38 | 0.914 |
| Pubis | 0.85±0.04 | 0.86±0.04 | 0.86±0.05 | 0.790 | 0.93±0.06 | 0.91±0.06 | 0.90±0.07 | 0.138 | 16.64±6.75 | 18.25±8.38 | 14.37±5.56 | 0.126 |

S1: a dataset of patients with PI-RADS score of 1-2 or biopsy-proven benign prostate hyperplasia (Dataset 1);

S2: a dataset of biopsy-proven PCa patients without bone metastases (Dataset 2);

S3: a dataset of biopsy-proven PCa patients with bone metastases (Dataset 3).

DSC: Dice similarity coefficient; HD: Hausdorff distance; VS: Volumetric similarity.

**Table S2.** Segmentation performance of pelvic bony structures among different vendors on DWI images

| Pelvic bony structures | DSC | | | *P* value | VS | | | *P* value | HD | | | *P* value |
| --- | --- | --- | --- | --- | --- | --- | --- | --- | --- | --- | --- | --- |
|  | V1 | V2 | V3 |  | V1 | V2 | V3 |  | V1 | V2 | V3 |  |
| Lumbar vertebra | 0.89±0.04 | 0.87±0.06 | 0.87±0.04 | 0.174 | 0.94±0.06 | 0.91±0.05 | 0.93±0.05 | 0.190 | 11.58±3.85 | 9.51±1.58 | 12.36±2.93 | 0.109 |
| Sacrococcyx | 0.89±0.03 | 0.87±0.04 | 0.86±0.04 | 0.054 | 0.96±0.03 | 0.96±0.03 | 0.94±0.04 | 0.139 | 12.80±4.68 | 12.20±2.40 | 16.42±5.81 | 0.150 |
| Ilium | 0.88±0.03 | 0.88±0.03 | 0.86±0.03 | 0.061 | 0.97±0.02 | 0.97±0.02 | 0.97±0.02 | 0.651 | 13.79±4.41 | 11.38±2.50 | 13.57±4.11 | 0.130 |
| Acetabulum | 0.85±0.04 | 0.86±0.03 | 0.84±0.03 | 0.369 | 0.94±0.05 | 0.95±0.04 | 0.94±0.05 | 0.878 | 17.17±6.41 | 15.64±4.88 | 17.38±5.82 | 0.654 |
| Femoral head | 0.91±0.04 | 0.89±0.05 | 0.88±0.05 | 0.207 | 0.95±0.04 | 0.96±0.03 | 0.94±0.05 | 0.569 | 9.57±3.28 | 7.62±1.68 | 8.23±1.43 | 0.054 |
| Femoral neck | 0.89±0.03 | 0.85±0.04 | 0.87±0.03 | 0.073 | 0.96±0.04 | 0.94±0.07 | 0.95±0.05 | 0.303 | 12.72±4.89 | 11.40±3.61 | 12.15±3.05 | 0.578 |
| Ischium | 0.86±0.03 | 0.86±0.04 | 0.85±0.04 | 0.532 | 0.93±0.05 | 0.91±0.05 | 0.93±0.05 | 0.355 | 18.22±7.03 | 16.67±7.43 | 17.81±6.36 | 0.762 |
| Pubis | 0.85±0.05 | 0.87±0.04 | 0.87±0.06 | 0.210 | 0.91±0.06 | 0.93±0.05 | 0.93±0.07 | 0.594 | 16.67±7.29 | 16.26±6.91 | 17.27±6.92 | 0.931 |

V1: 3.0 T Discovery;V2: 3.0T Achieva; V3: 3.0 T Intera

DSC: Dice similarity coefficient; HD: Hausdorff distance; VS: Volumetric similarity.

**Table S3.** Segmentation performance of pelvic bony structures among different datasets on T1WI-IP images

| Pelvic bony structures | DSC | | | *P* value | VS | | | *P* value | HD | | | *P* value |
| --- | --- | --- | --- | --- | --- | --- | --- | --- | --- | --- | --- | --- |
|  | S1 | S2 | S3 |  | S1 | S2 | S3 |  | S1 | S2 | S3 |  |
| Lumbar vertebra | 0.87±0.05 | 0.90±0.04 | 0.91±0.04 | 0.443 | 0.97±0.03 | 0.94±0.09 | 0.97±0.04 | 0.292 | 9.45±3.01 | 11.36±5.25 | 11.34±5.59 | 0.403 |
| Sacrococcyx | 0.87±0.04 | 0.88±0.04 | 0.90±0.03 | 0.458 | 0.98±0.02 | 0.97±0.02 | 0.97±0.02 | 0.422 | 8.85±3.92 | 10.84±5.35 | 9.44±4.58 | 0.442 |
| Ilium | 0.87±0.03 | 0.88±0.02 | 0.88±0.04 | 0.058 | 0.99±0.01 | 0.98±0.03 | 0.99±0.01 | 0.328 | 8.06±4.21 | 8.93±2.51 | 8.69±2.62 | 0.719 |
| Acetabulum | 0.86±0.04 | 0.84±0.04 | 0.85±0.05 | 0.068 | 0.96±0.03 | 0.95±0.03 | 0.97±0.04 | 0.357 | 12.80±5.31 | 15.15±7.39 | 12.08±4.09 | 0.288 |
| Femoral head | 0.90±0.04 | 0.92±0.04 | 0.88±0.05 | 0.224 | 0.97±0.02 | 0.97±0.02 | 0.97±0.02 | 0.834 | 4.54±1.54 | 5.45±1.84 | 4.53±1.05 | 0.151 |
| Femoral neck | 0.88±0.03 | 0.89±0.03 | 0.87±0.05 | 0.071 | 0.99±0.02 | 0.99±0.01 | 0.96±0.08 | 0.105 | 9.56±5.56 | 5.16±1.55 | 9.92±8.52 | 0.152 |
| Ischium | 0.86±0.04 | 0.86±0.03 | 0.85±0.04 | 0.156 | 0.94±0.05 | 0.97±0.02 | 0.97±0.02 | 0.050 | 17.18±6.77 | 16.26±5.04 | 16.15±6.90 | 0.867 |
| Pubis | 0.85±0.04 | 0.86±0.04 | 0.86±0.05 | 0.113 | 0.95±0.04 | 0.94±0.05 | 0.93±0.07 | 0.639 | 9.91±4.49 | 11.77±4.85 | 10.44±4.55 | 0.510 |

S1: a dataset of patients with PI-RADS score of 1-2 or biopsy-proven benign prostate hyperplasia (Dataset 1);

S2: a dataset of biopsy-proven PCa patients without bone metastases (Dataset 2);

S3: a dataset of biopsy-proven PCa patients with bone metastases (Dataset 3).

DSC: Dice similarity coefficient; HD: Hausdorff distance; T1WI-IP : T1W images obtained using the Dixon technique with in-phase; VS: Volumetric similarity.

**Table S4.** Segmentation performance of pelvic bony structures among different vendors on T1WI-IP images

| Pelvic bony structures | DSC | | | *P* value | VS | | | *P* value | HD | | | *P* value |
| --- | --- | --- | --- | --- | --- | --- | --- | --- | --- | --- | --- | --- |
|  | V1 | V2 | V3 |  | V1 | V2 | V3 |  | V1 | V2 | V3 |  |
| Lumbar vertebra | 0.93±0.03 | 0.91±0.04 | 0.93±0.02 | 0.174 | 0.97±0.03 | 0.92±0.10 | 0.97±0.03 | 0.056 | 10.60±4.60 | 10.61±5.74 | 10.83±3.29 | 0.993 |
| Sacrococcyx | 0.94±0.02 | 0.93±0.02 | 0.93±0.03 | 0.054 | 0.98±0.02 | 0.97±0.02 | 0.98±0.03 | 0.688 | 9.12±4.10 | 10.56±5.24 | 9.40±4.77 | 0.629 |
| Ilium | 0.94±0.02 | 0.94±0.03 | 0.95±0.02 | 0.061 | 0.99±0.01 | 0.98±0.03 | 0.99±0.01 | 0.724 | 8.31±2.52 | 8.41±2.02 | 8.98±5.44 | 0.837 |
| Acetabulum | 0.90±0.03 | 0.90±0.03 | 0.90±0.03 | 0.369 | 0.96±0.04 | 0.96±0.03 | 0.97±0.03 | 0.915 | 14.90±5.69 | 10.16±4.44 | 12.82±5.47 | 0.053 |
| Femoral head | 0.93±0.04 | 0.96±0.02 | 0.95±0.03 | 0.207 | 0.97±0.02 | 0.98±0.01 | 0.97±0.03 | 0.171 | 4.65±1.37 | 5.13±1.92 | 4.63±1.35 | 0.605 |
| Femoral neck | 0.94±0.04 | 0.95±0.02 | 0.95±0.02 | 0.073 | 0.97±0.07 | 0.99±0.01 | 0.99±0.02 | 0.391 | 6.96±6.17 | 8.28±6.98 | 11.68±9.73 | 0.184 |
| Ischium | 0.90±0.04 | 0.91±0.03 | 0.90±0.03 | 0.532 | 0.96±0.04 | 0.95±0.04 | 0.96±0.03 | 0.701 | 16.21±6.42 | 18.21±6.25 | 15.60±6.18 | 0.520 |
| Pubis | 0.87±0.03 | 0.86±0.04 | 0.90±0.04 | 0.210 | 0.95±0.05 | 0.91±0.06 | 0.95±0.03 | 0.159 | 10.99±4.31 | 10.30±4.15 | 10.17±5.68 | 0.846 |

V1: 3.0 T Discovery;V2: 3.0T Achieva; V3: 3.0 T Intera

DSC: Dice similarity coefficient; HD: Hausdorff distance; T1WI-IP : T1W images obtained using the Dixon technique with in-phase; VS: Volumetric similarity.
